# The Combined Effects of Ethylene and MeJA on Metabolic Profiling of Phenolic Compounds in *Catharanthus roseus* Revealed by Metabolomics Analysis

**DOI:** 10.3389/fphys.2016.00217

**Published:** 2016-06-07

**Authors:** Jia Liu, Yang Liu, Yu Wang, Zhong-Hua Zhang, Yuan-Gang Zu, Thomas Efferth, Zhong-Hua Tang

**Affiliations:** ^1^The Key Laboratory of Plant Ecology, Northeast Forestry UniversityHarbin, China; ^2^Department of Pharmaceutical Biology, Institute of Pharmacy and Biochemistry, Johannes Gutenberg UniversityMainz, Germany

**Keywords:** *Catharanthus roseus*, phenolic compounds, ethylene, methy jasmonate, non-targeted metabolomics

## Abstract

Phenolic compounds belong to a class of secondary metabolites and are implicated in a wide range of responsive mechanisms in plants triggered by both biotic and abiotic elicitors. In this study, we approached the combinational effects of ethylene and MeJA (methyl jasmonate) on phenolic compounds profiles and gene expressions in the medicinal plant *Catharanthus roseus*. In virtue of a widely non-targeted metabolomics method, we identified a total of 34 kinds of phenolic compounds in the leaves, composed by 7 C6C1-, 11 C6C3-, and 16 C6C3C6 compounds. In addition, 7 kinds of intermediates critical for the biosynthesis of phenolic compounds and alkaloids were identified and discussed with phenolic metabolism. The combinational actions of ethylene and MeJA effectively promoted the total phenolic compounds, especially the C6C1 compounds (such as salicylic acid, benzoic acid) and C6C3 ones (such as cinnamic acid, sinapic acid). In contrast, the C6C3C6 compounds displayed a notably inhibitory trend in this case. Subsequently, the gene-to-metabolite networks were drawn up by searching for correlations between the expression profiles of 5 gene tags and the accumulation profiles of 41 metabolite peaks. Generally, we provide an insight into the controlling mode of ethylene-MeJA combination on phenolic metabolism in *C. roseus* leaves.

## Introduction

*Catharanthus roseus* (Madagascar periwinkle) is a medicinal plant producing about more than 150 different terpenoid indole alkaloids (TIAs). Among the many pharmaceutically important TIAs are vinblastine and vincristine, the well-known anticancer agents that have been long in clinical use (van Der Heijden et al., 2004). Nevertheless, report has also suggested that *C. roseus* contains a wide spectrum of phenolic compounds besides TIAs (Filippini et al., 2003; Mustafa and Verpoorte, 2007; Ferreres et al., 2008), such as flovonoids, caffeic acid, benzoic acid, and cinnamic acid derivatives. These natural products are often involved in signaling pathways triggered as a defense mechanism against biotic and abiotic stresses (Demkura et al., 2010). Furthermore, they contribute to human health by exerting various biological activities including antioxidant, antibacterial and anticancer (Valentao et al., 2001; Sousa et al., 2008).

**Figure 1:**
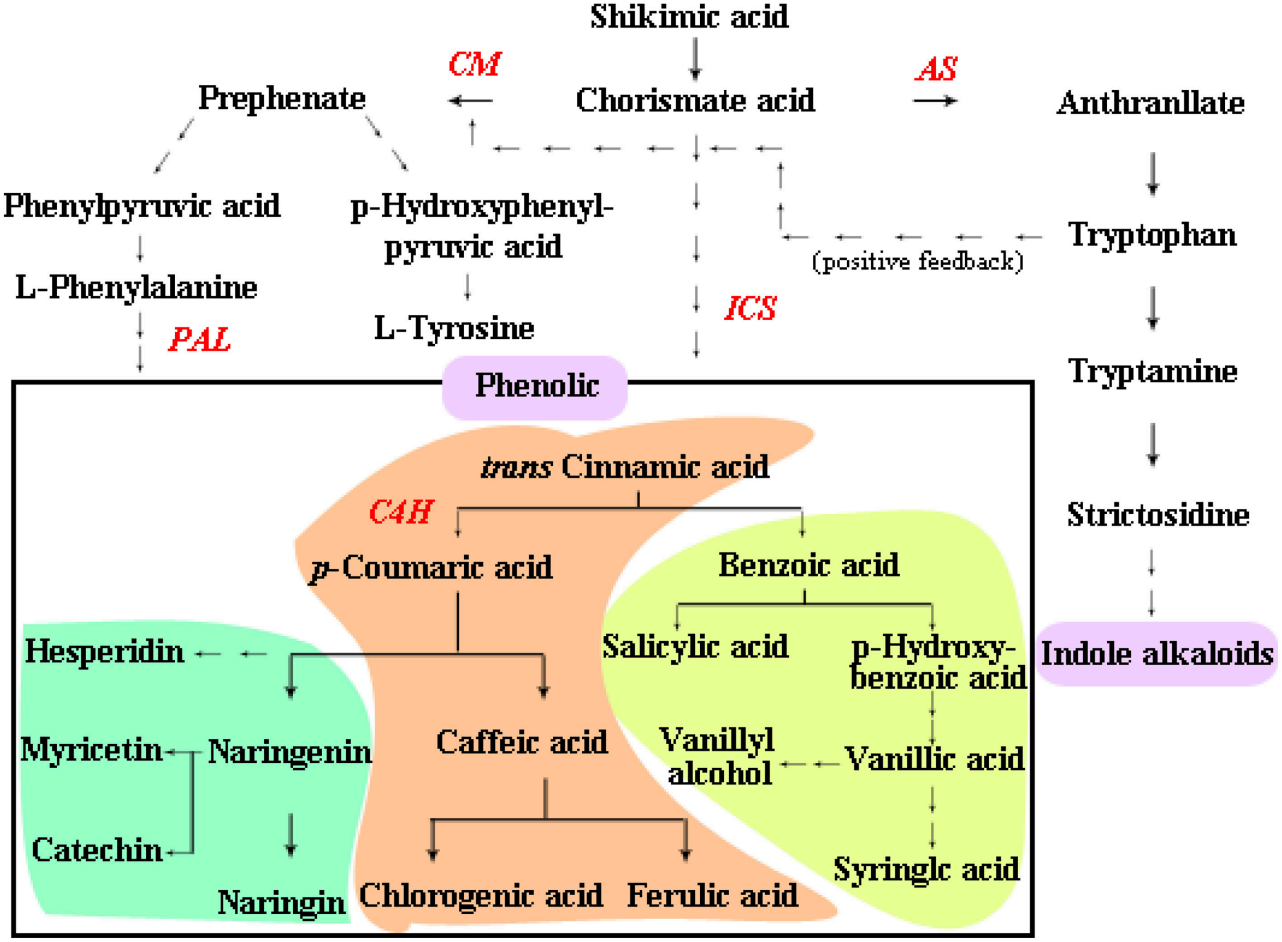
**The biosynthetic pathway of phenolic compounds**. *Green region* indicates C6C3C6-compounds; *Orange region* indicates C6C3-compounds; *Yellow region* indicates C6C1-compounds; *Purple region* indicates a class of metabolites. *Red* indicates the key enzymes, the following abbreviations are used for different pathway enzymes: *CM* (chorismate mutase), *ICS* (isochorismate synthase), *PAL* (phenylalanine ammonia-lyase), *C4H* (*trans*-cinnamate 4-monooxygenase), and *AS* (amyrin synthase). The solid-line arrow represents one-step reaction, and dashed-line arrow means multi-steps reactions.

Phenolics are the most widely distributed metabolites that are involved in the interactions between biology and environments (Garcia-Calderon et al., 2015). In plants, phenolic compounds are synthesized via the phenylpropanoid pathway that begins with the conversion of phenylalanine to cinnamic acid by phenylalanine ammonia lyase (*PAL*) and hence also compete with the indole alkaloid biosynthesis for common precursor chorismate (Ferreres et al., 2008). Based on the so far gathered knowledge on spatial and temporal complexities associated with phenolic pathway in *C. roseus*, the involvement of at least 20 coordinately regulated enzymatic steps occurring in different tissues types has been implicated, such as *CM* (chorismate mutase), *ICS* (isochorismate synthase), *PAL* (phenylalanine ammonia-lyase), and *C4H* (*trans*-cinnamate 4-monooxygenase) (Mustafa and Verpoorte, 2005; Ferreres et al., 2011). Phenolic compounds are referred to as cyclic compounds with exchangeable hydroxyl groups and are classified based on the number and binding position of convertible hydroxyl groups on the aromatic chain into three major groups: simple phenolic compounds (mass compounds have a C6C1 carbon skeleton, usually with a carboxyl group attached to the aromatic ring, such as benzoic acid), phenolic acid derivatives (derived from phenylalanine, having a C6C3 carbon skeleton, such as caffeic acid), and flavonoids (compounds having C6C3C6 carbon skeleton such as flavonoids and isoflavonoids; Dixon, 2001; Mustafa and Verpoorte, 2007). So far, around 5000 phenolic compounds have been identified and their contents fluctuate depending on environmental conditions and plant species (Mustafa and Verpoorte, 2007; Simirgiotis et al., 2015; Vos et al., 2015). The accumulation of phenolics may also affect other secondary metabolite pathways including alkaloid pathways, since plant defense is a complex system (Mustafa and Verpoorte, 2007; Ferreres et al., 2008; Figure 1).

Identification and structural characterization of phenolic compounds has been well developed and achieved using LC-MS/MS in different plant species (Ferreres et al., 2004; Lin and Harnly, 2007; Dong et al., 2014). The metabolic profiling analysis of phenolic compounds in some plant extracts showed that the *C. roseus* extracts contained the highest amount of a C6C3 hydroxytyrosol and a C6C1 gallic acid when compared to 26 other plant extracts analyzed. Other phenolics detected in this plant were ferulic acid and vanillic acid. No flavonoids were detected in this study (Proestos et al., 2005). Filippini et al. developed a stable callus of *C. roseus* producing anthocyanins by continuous cell-aggregate selection (Filippini et al., 2003). Similar anthocyanins were identified by LC-MS/MS metabolic profiling. They were also identified as 3-*O*-glucosides and 3-*O*-(6-*O*-*p*-coumaroyl) glucosides of petunidin, malvidin, and hirsutidin. Moreover, three caffeoylquinic acids and fifteen flavonol glycosides were identified by the same level of technology (Ferreres et al., 2008). As mentioned above, the potential of developing a platform for qualitative analysis of phenolic compounds has been highlighted in several studies. Therefore, we approached the comprehensively analysis of phenolic compounds located in *C. roseus* leaves using this method.

The phytohormones ET and methyl jasmonate (MeJA) were reported to elicit secondary metabolites such as alkaloids and phenolic compounds regulating plant growth and adaptation (Demkura et al., 2010; Vanstraelen and Benkova, 2012; Wasternack and Hause, 2013; Cocetta et al., 2015; Pozo et al., 2015). ET acts as an intermediate signaling molecule in elicitor-induced terpenoid indole alkaloids accumulation (Papon et al., 2005; Pan et al., 2015). ET is also able to induce flavonol accumulation alone or interact with auxin (Lewis et al., 2011; Watkins et al., 2014). The addition of MeJA to *C. roseus* hairy root cultures increased the yields of ajmalicine, serpentine, lochnericine, and hörhammericine (Rijhwani and Shanks, 1998). MeJA also tightly modulated phenolic metabolism and gene expression in blueberry (Cocetta et al., 2015), under solar ultraviolet B radiation (Demkura et al., 2010), or in broccoli sprouts (Carvacho et al., 2014). In some cases, concomitant activation of JA and ET response pathways is required for induction of plant defensive gene, such as PDF1.2 (Penninckx et al., 1998). These reports provided critical insight into the roles of ET or MeJA in the modulation of alkaloids and phenolic compounds, however, their combined effects on phenolic profiles remain into investigation. Here, we mainly focused on the combinational effects of ET and MeJA on phenolic compound profiles and gene expressions in *C. roseus* leaves in virtue of metabolomics approach.

## Results

### The combined effects of ET and MeJA on the metabolic profiles of total phenolic compounds

**Figure 2:**
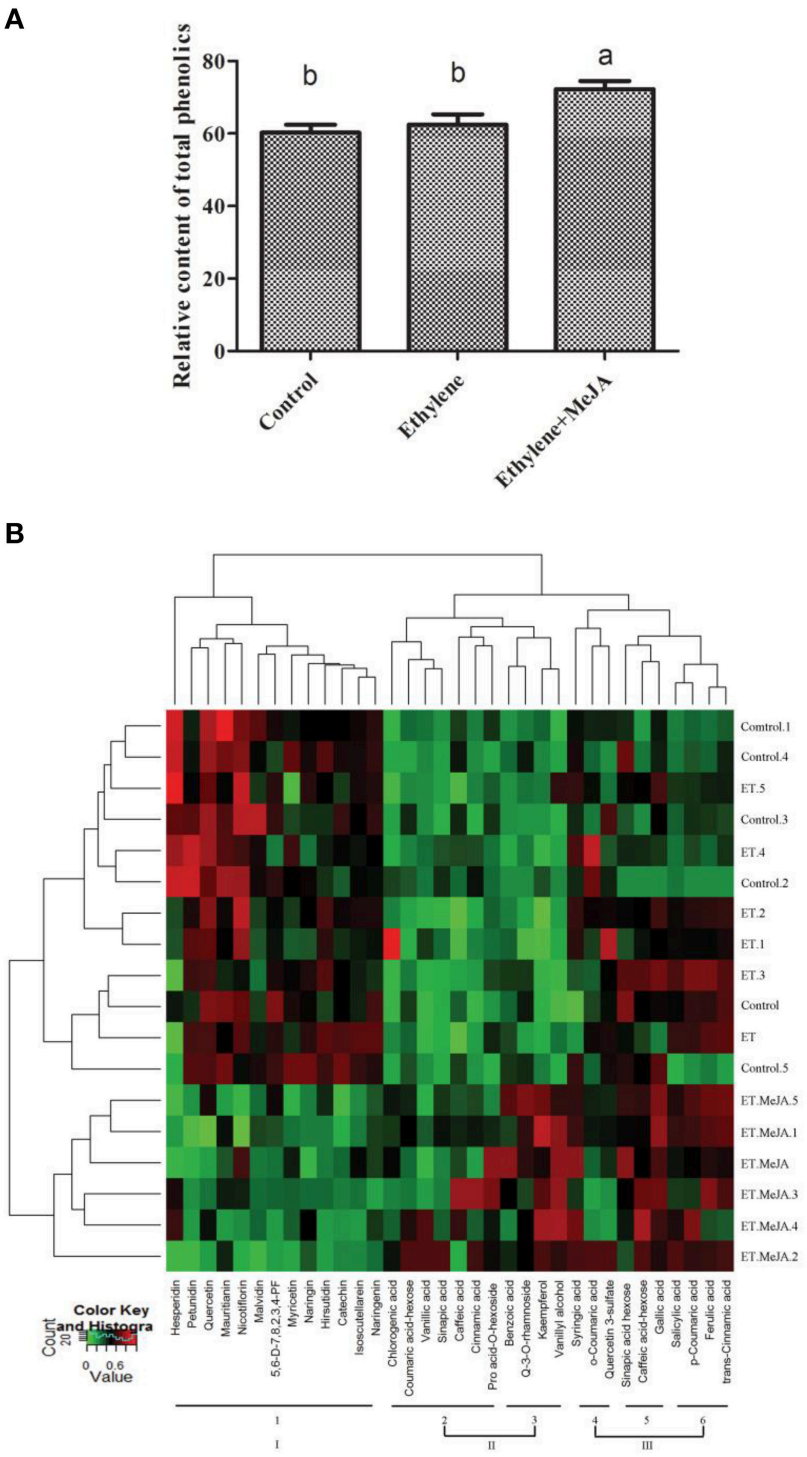
**The combined effects of ET and MeJA on the metabolic profiles of total phenolic compounds. (A)** Ethlyene and/or jasmonate enhance the total phenolic production in *C. roseus* leaves. CK: control, ET: ET, MeJA: jasmonate. **(B)** Heat map visualization of relative differences of phenolics in different treatment. Data of the relative content value of each phenolic were normalized to complete linkage hierarchical clustering. Each phenolic is visualized in a single column and each hormone type is represented by a single row, including six repeat. Abbreviations: 5.6-D-7,8,2,3,4-PF (5,6′-dihydroxy-7,8,2′,3′,4′-Pentamethoxyflavone), Pro acid-*O*-hexoside (Protocatechuic acid-*O*-hexoside), and Q-3-*O*-rhamnoside (Quercetin-3-*O*-rhamnoside). *Red* indicates high abundance, whereas low relative phenolics are green. Each bar represents the mean ± S. E. M., *n* = 6.

Using LC-QTOF-MS/MS, we developed a novel widely non-targeted metabolomics method for the comprehensive profiling analysis of phenolic compounds in *C. roseus*. According to the identity check based on raw data and the features of peaks, the target masses of candidate metabolites identified in the profiling process were searched over a narrow + 5 ppm mass window in HMDB, METLIN, and KEGG databases. A total of 34 phenolics that belong to three categories (7 C6C1-, 11 C6C3-, and 16 C6C3C6 compounds; Table 1), and seven other metabolites were identified in our study (Table S2). The relatively level of total phenolic compounds significantly increased (*p* < 0.01) in plants treated with ET and MeJA compared with those under conditions of control or plus ET only (Figure 2A).

**Table 1:** **Structure of identified phenolic compounds**.

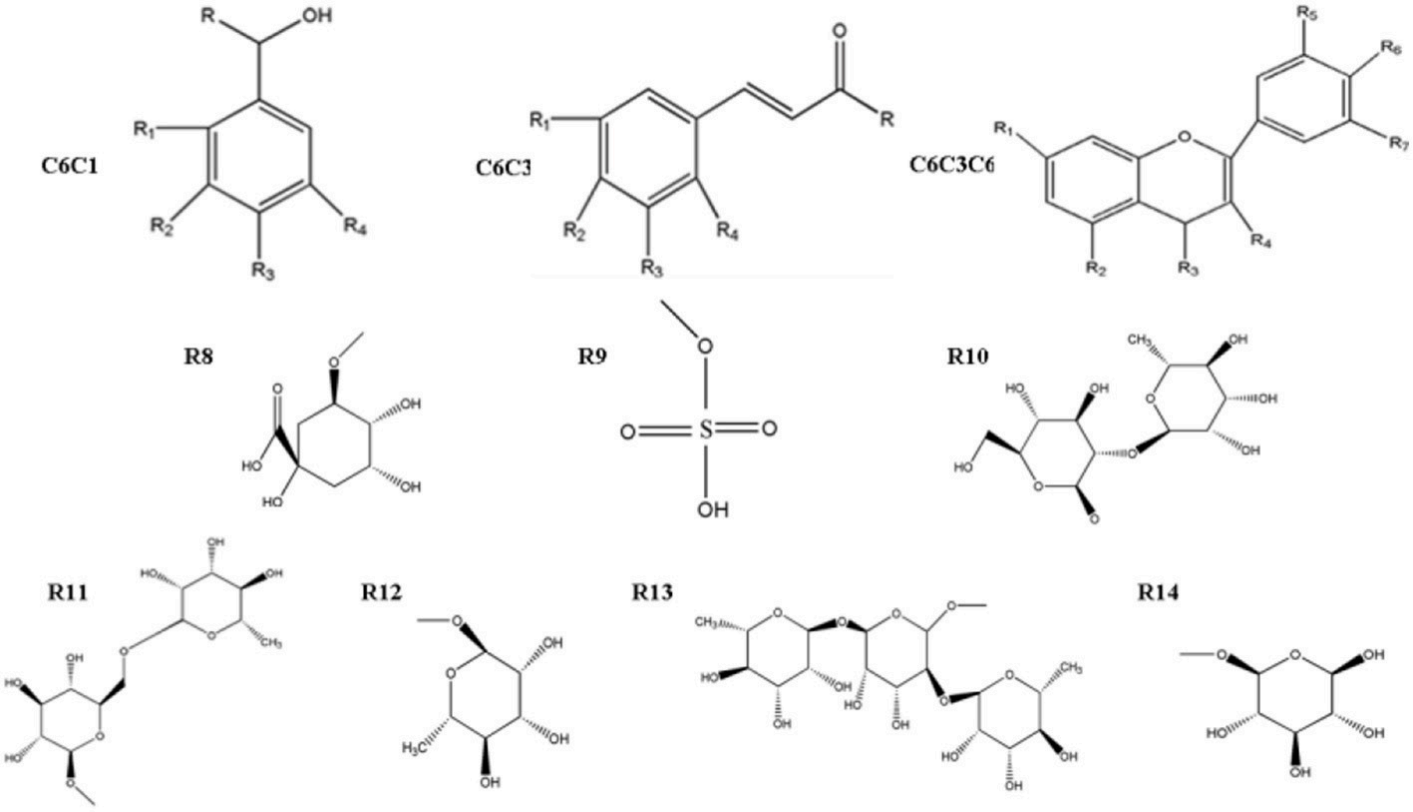									
**Structure**	**Derivates**	**R**	**R**_1_	**R**_2_	**R**_3_	**R**_4_	**R**_5_	**R**_6_	**R**_7_
C6C1	Salicylic acid	= O	−OH						
	Vanillic acid	= O		−OCH_3_	−OH				
	Vanillyl alcohol			−OCH_3_	−OH				
	Syringic acid	= O		OCH_3_	−OH	−OCH_3_			
	Gallic acid	= O		−OH	−OH	−OH			
	Benzoic acid	= O							
C6C3	*Trans*-cinnamric acid	−OH							
	Cinnamic acid	−OH							
	*p*-Coumanic acid	−OH		−OH					
	*o*-Cumamic acid	−OH				−OH			
	Caffeic acid	−OH	−OH	−OH					
	Ferulic acid	−OH	−OCH_3_	−OH	−OCH_3_				
	Sinapic acid		−OH	−OH					
	Chlorogenic acid	R_8_	−OH	−OH					
C6C3C6	Catechin	−OH	−OH	−OH		−OH	−OH		
	Naringenin		−OH	−OH	−O			−OH	
	Hirsutidin		−OCH_3_	O^−^		−OH	−OCH_3_	−OH	−OCH_3_
	Myricetin		−OH	−OH	O	−OH	−OH	−OH	−OH
	Kaempferol	−OH	−OH	−OH	= O			−OH	
	Isoscutellarein	−OH	−OH	−OH	= O			−OH	
	Quercetin		−OH	−OH	= O	−OH	−OH	−OH	
	Quercetin-3-sulfate		−OH	−OH	= O	R_9_	−OH	−OH	
	Malvidin		O^−^	−OH	–	OH	−OCH_3_	−OH	−OCH_3_
	Naringin		R_10_	−OH	= O			−OH	
	Hesperidin		R_11_	−OH	= O		−OH	−OCH_3_	
	Nicotiflorin		−OH	−OH	= O	R_11_		−OH	
	Petunidin		R_13_	−OH	= O	R_11_	−OH	−OH	
	Quercetin-3-*o*-rhamnoside		−OH	−OH	= O	R_12_		−OH	−OH
	Mauritianin		−OH	−OH	= O	R_14_		−OH	

Subsequently, the visualization of the phenolic compounds profiles was performed by hierarchical cluster analysis (HCA; Figure 2B). The results showed that accumulation of phenolic metabolites displayed a clear variation in terms of their abundance upon different treatment. Co-treatment of both hormones contained the highest levels of most phenolics, followed by treatment with ET alone. Based on their responses to different treatment, phenolic profiles could be clearly grouped into three main clusters with six subclusters. Phenolics in cluster I were mainly represented by C6C3C6 compounds with lower levels detected in ET and MeJA, such as hesperidin, petunidin, quercetin, mauritianin, nicotiflorin, malvidin, 5,6-D-7,8,2,3,4-PF, myricetin, naringin, hirsutidin, catechin, isoscutellarein, and naringenin. Major C6C3-(chlorogenic acid, coumaric acid-hexose, sinapic acid, caffeic acid, and cinnamic acid) and C6C1-(vanillic acid and pro acid-O-hexoside) compounds were tightly grouped in subcluster 2. These phenolic displayed higher levels upon the combined action of ET and MeJA compared to the controls. In addition, those in subcluster 3 were mainly represented by compound C6C1-(benzoic acid and vanillyl alcohol) and C6C3C6-(Q-3-O-rhamnoside and kaempferol), also displaying highest levels under the combined condition. Three C6C3-(*p*-coumaric acid, ferulic acid, and *trans*-cinnamic acid) and one C6C1-(salicylic acid) in subcluster 6 showed ET-specific accumulation pattern. However, *o*-coumarica acid, quercetin 3-sulfate, and sinapic acid hexose in subcluster 4 and 5, respectively, showed little sensitivity to ET or/and MeJA, which subcluster 4, 5, and 6 belonging to the last cluster III. The results showed that ET and MeJA combination could mainly promote C6C1- and C6C3-metabolites accumulation, except for C6C3C6 compounds.

### The combined effects of ET and MeJA on C6C1-, C6C3-, and C6C3C6-type compounds

**Figure 3:**
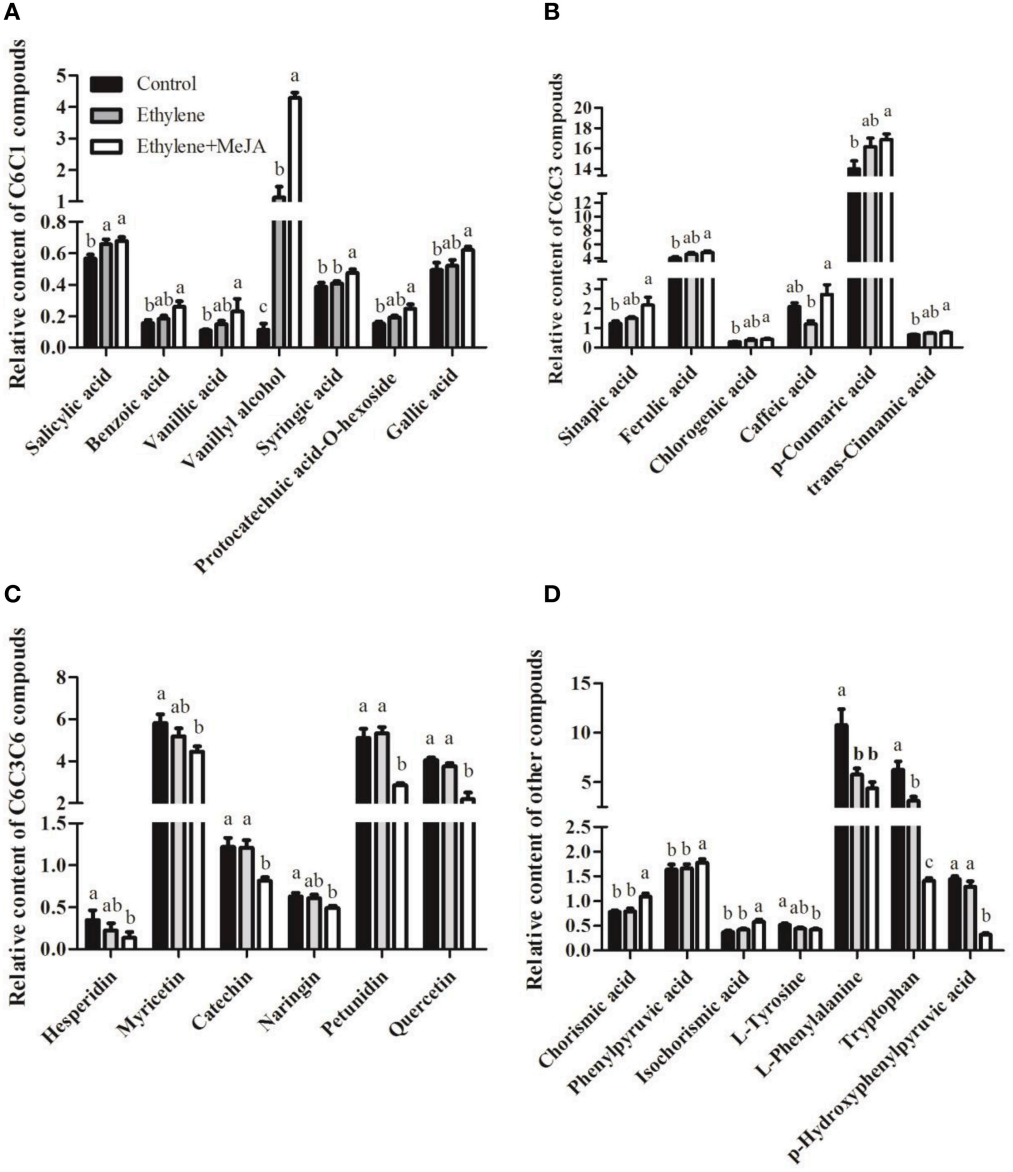
**The combined effects of ET and MeJA on C6C1-, C6C3-, and C6C3C6-type compounds. (A)** The relative content of the structure formula C6C1. **(B)** Six major C6C3-phenolic relative contents. **(C)** Six major C6C3C6-phenolic relative contents. **(D)** Other metabolites relative contents. Other C6C3- and C6C3C6-compounds are given in Figure S1. Each bar represents the mean ± S. E. M., *n* = 6.

Plant phenolic compounds are synthesized via the chorismate and can be majorly divided into three types, including C6C1, C6C3, and C6C3C6. Their specific responses were further clarified. For most of the C6C1- and C6C3-type compounds, there was a significant promotion by the combinational action of ET and MeJA compared with the treatments with control solution and plus ET alone (Figures 3A,B). Among these compounds, vanillyl alcohol in the leaves was found to be elevated 50 times in the presence of ET and MeJA compared with the control case. In contrast, the C6C3C6-type compounds displayed an inhibited accumulation upon ET plus MeJA compared with the cases upon ET alone or control solution (Figure 3C). Furthermore, parts of the intermediates in the phenolic or TIA biosynthetic pathway were identified here, including chorismic acid, phenylpyruvic acid, isochorismic acid, L-tyrosine, L-phenylalaine, tryptophan, and *p*-hydroxyphenylpyruvic acid. The actions of ET plus MeJA obviously promoted the first three compounds and inhibited the remaining four ones, respectively (Figure 3D). The further analysis of other major C6C3- and C6C3C6 compounds identified in our research showed that cinnamic acid (C6C3) was largely promoted and most of the C6C3C6 compounds were decreased by the combination of ET and MeJA (Figure S1).

### The combined effects of ET and MeJA on the gene expression responsible for phenolic or alkaloid biosynthesis

**Figure 4:**
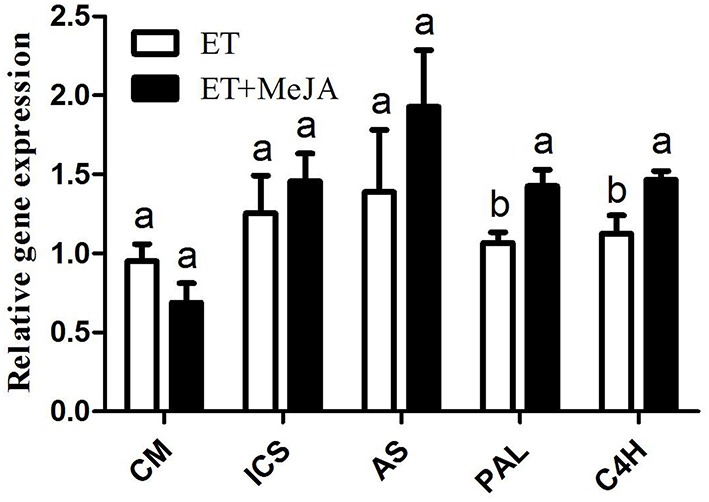
**Differential expression of genes in leaves treated with either ET alone or ET plus MeJA**. Each treatment is compared with CK: if greater than 1, it means promotion; if lower than 1, it means suppression.

Other than the metabolic analysis, the gene expressions responsible for phenolic biosynthesis (CM: chorismate mutase; ICS: isochorismate synthase; PAL: phenylalanine ammonia lyase; C4H: cytochrome P450 hydroxylation) were compared with that for indole alkaloid pathway (AS: anthranilate synthase). The treatment with ET precursor ACC alone enhanced the expression levels of *ICS, AS, PAL*, and *C4H* genes than the control treatment. The inclusion of MeJA plus ET further significantly improved the expression levels of *PAL* and *C4H* genes when compared with the treatment with ET alone (Figure 4). In this case, the *CM, ICS, and AS* gene expressions were not obviously changed.

### Integrated response analysis of genes—metabolites network

**Figure 5:**
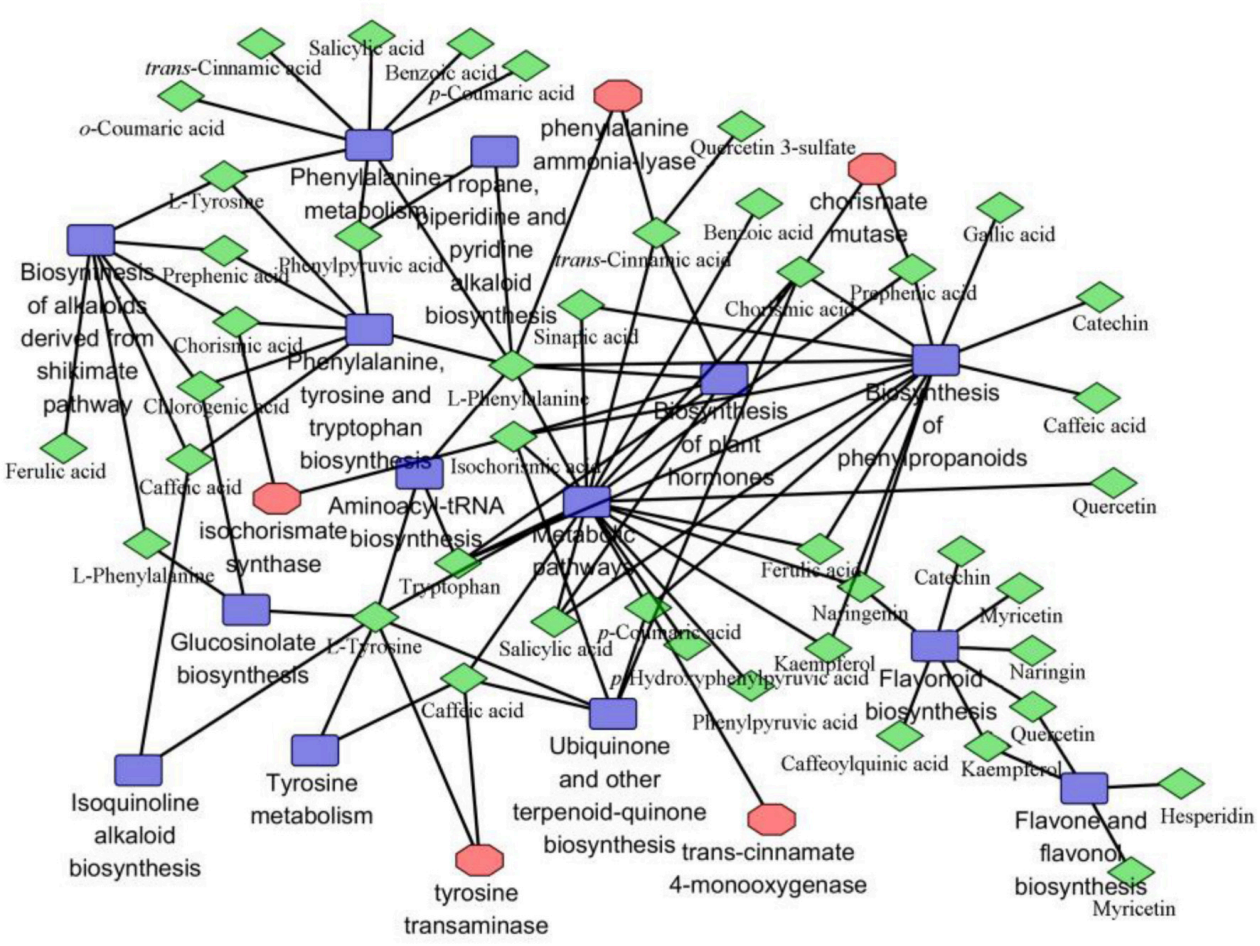
**Gene and metabolite network analysis in elicited *C. roseus* leaves**. Metabolites are represented by green-diamond, genes by red-rolygon, and related pathway by blue-square. Correlation network is composed of 34 phenolic compounds combine with five genes and other relevant metabolites. Metabolites with KEGG from the merged data set were mapped to KEGG and MBRole reference pathway, and interaction networks were generated in Cytoscape, *p* > 0.005.

To facilitate access to the total metabolic profiles, the metabolites, genes and corresponding pathways were imported into Cytoscape for visualization of the network models. The intersection of the networks was done using the advanced network merge function in Cytoscape. Altered metabolites with KEGG from the merged data set were mapped to KEGG reference pathways, and interaction networks were generated in Cytoscape. As seen in Figure 5, the association network of differentially expressed metabolites using Cytoscape was constructed. Modes of action are shown in different colors and shapes. Networks are represented as graphs, where the green diamond, red polygon, and blue square nodes represent metabolites, genes, and related pathways detected, respectively. These closely connected and differentially expressed metabolites are regarded as the target signatures. The significantly overrepresented categories indicated that this emergent new and advanced network was composed by metabolites, genes, and related pathways. Fortunately, for the greater part of the identified metabolites and genes could be included in a network together through indirect interaction or only one intermediate partner. However, a small portion of phenolic derivatives were not found their compound identifiers (IDs) from KEGG, so these could not be included, such as protocatechuic acid-*O*-hexoside, coumaric acid-hexose, sinapic acid-hexose, quercetin-3-*O*-rhamnoside, and caffeic acid-hexose. The regulatory patterns of phenolic compounds presented here provide evidence that metabolites are actively involved in multifunctional pathways, and these insights help us to better understand the mechanisms underlying their responses to ET plus MeJA.

## Discussions

In this study, we utilized the widely non-targeted metabolomics method for the direct chemical screening of phenolic compounds in the medicinal plants *C. roseus*. We identified a total of 34 phenolic compounds, mainly including C6C1-, C6C3-, and C6C3C6-type compounds. The result is consistent with previous reports that *C. roseus* contains the C6C1-, C6C3-, and C6C3C6 compounds (Dixon, 2001; Choi et al., 2004; Mustafa and Verpoorte, 2005, 2007). Our further analysis showed that there were 7, 11, and 16 kinds of compounds, respectively, belonging to C6C1-, C6C3-, and C6C3C6-type. MBRole performs enrichment analysis of a number of annotations of diverse nature coming from KEGG, which are annotated with their associated pathways, enzymes and chemical groups, as well as other interactions (Chagoyen and Pazos, 2011). However, a number of phenolic derivatives did not find these corresponding IDs, such as protocatechuic acid-*O*-hexoside, coumaric acid-hexose, sinapic acid-hexose, quercetin-3-*O*-rhamnoside, and caffeic acid-hexose. In addition, the results (Table S3) contain the list of annotations over-represented in the input set with respect to the background set and their associated *P* < 0.005 (Zhang et al., 2013). Every pathway listed in the MBRole data set with at least on metabolite identified by the MS associated with that pathway was noted, and the MS-identified metabolites were crossly listed with the pathway. Each metabolite could belong to multiple pathways (Figure 5).

The phenolic compounds play diverse and important functions in plant development and defense as reactive oxygen species scavengers, disease resistance inducer (Moreno et al., 1994; Murphy et al., 2000; Mustafa and Verpoorte, 2007; Watkins et al., 2014). In our study, C6C1-, C6C3-, and C6C3C6-compounds comprised 3.58, 51.81, and 44.60% of total phenolic compounds, respectively. It suggested that C6C3C6 compounds are mainly involved in plant flavonoid biosynthesis, functioning as filters against ultraviolet irradiation (Trantas et al., 2015). In contrast, the C6C1 compounds might mainly play an important role as signaling molecules, such as the BA (benzoic acid)-derivatives SA (salicylic acid; Cheynier et al., 2013). Because, the biosynthesis of C6C1 compounds are reported to be most strictly induced by biotic elicitors (Mustafa and Verpoorte, 2007) and these low-molecular weight phenolics occur universally in higher plants (Cheynier et al., 2013). We found that the elevated compounds in the leaves after the co-treatment mainly belonged to either the C6C1 or C6C3-compounds. Evidence is accumulating that components from SA-, JA-, and ET-dependent defense pathways can affect each other's signaling (van Wees et al., 2000). The synergistic cross-talk between JA and ET is known to occur preferentially for the response to necrotrophic pathogens (Wasternack and Hause, 2013) and activate JA-dependent plant defenses against herbivores via alkaloid biosynthesis (Onkokesung et al., 2010). However, SA and its functional analogs suppress JA-dependent defense gene expression, possibly through the inhibition of JA synthesis and action (van Wees et al., 2000; Vos et al., 2015). Conversely, other reports showed that ET and MeJA were shown to stimulate SA action (van Wees et al., 2000). We found that the exogenous ET plus MeJA slightly promoted accumulations of salicylic acid (SA) and its derivatives benzoic acid, isochorismic acid, whereas largely enhanced levels of vanillic acid, vanillyl alcohol, and syringic acid than did ET alone (Figures 3A,B). The compound syringic acid and its precursors (vanillic acid and vanillyl alcohol) compete with SA for benzoic acid as substrate (Mustafa and Verpoorte, 2007), thus, we proposed that ET-JA crosstalk mainly antagonized SA *in vivo* synthesis and bring carbon flux to vanillic acid accumulation. However, our further clustering analysis found that SA was induced in an ET-specific way, suggesting that SA interplays synergistically with ET.

Our clustering results showed the C6C3 branch pathway metabolites, namely *p*-coumaric acid, ferulic acid, and *trans*-cinnamic acid, were grouped together with SA. These phenylpropanoids are derived from phenylalanine catalyzed by PAL enzyme (Mustafa and Verpoorte, 2007; Cheynier et al., 2013). Besides the precursors of C6C1 compounds, the above phenylpropanoids are also precursors of other phenolics, which in many plants act as phytoalexiins or phytoanticipins, such as flavonoids, isoflavonoids (Dixon, 2001) or as physical barrier against pathogen attack, such as lignin (Mustafa and Verpoorte, 2007). Activation of PAL is often considered as a hallmark for elicitation of SAR (systemic acquired resistance) in plants and SA is a necessary and sufficient signal for SAR (Clarke et al., 2000; van Wees et al., 2000). We found that ET alone or plus MeJA increased *PAL* transcripts and effectively decreased L-phenylalanine level, suggesting SA-dependent SAR process was induced. ET and MeJA are also implicated in induced systemic resistance (ISR; van Wees et al., 2000). ISR pathway may result in induction of different pathways leading to the production of phenolic compounds and/or other secondary metabolites (Glazebrook et al., 2003; Mustafa and Verpoorte, 2007). In contrast to C6C1 and C6C3 compounds, almost all the C6C3C6-metabolites displayed inhibitory accumulation after treatment with ET plus MeJA (Figure 3C and Figure S1B). The biosynthetic pathway of C6C3C6 compounds lead to productions of flavonoids (flavonols) and isoflavonoids (Mustafa and Verpoorte, 2007; Mouradov and Spangenberg, 2014). In vegetative tissues, anthocyanins and other flavonoids usually accumulate transiently, as a plastic response to biotic or abiotic stressors (Del Valle et al., 2015). For example, IAA and ET were able to regulate flavonol biosynthesis through distinct signaling networks and quercetin is revealed to be the flavonol that modulates basipetal auxin transport (Lewis et al., 2011). Our result is consistent with this report, because the reduced quercetin was caused by ET plus MeJA, whilst not by ET alone.

Other than phenolic compounds, a serial of intermediates in the biosynthetic pathway of phenolic and alkaloids were identified in this study (Figure 3D). Among them, the chorismate is a critical substrate for multiple enzymes, such as CM, ICS, and AS, resulting in different secondary metabolic pathway and carbon flux (Mustafa and Verpoorte, 2007; Van Lanen et al., 2008). The restrained expression of *CM* gene in our transcriptional analysis paralleled with the decreased levels of L-phenylalanine and L-tyrosine. In contrast, the *ICS* and *AS* expressions were slightly activated in this case. This evidence suggests that ET and MeJA prefer to elicit benzoic acid-derived phenolic compounds (C6C1), for example, vanillyl alcohol (50-fold increase) in our study. Our subsequent correlation analysis showed that the C6C1 compounds correlated positively with their precursors including chorismate acid and isochorismic acid, but showed a negative correlation with the tryptophan (Figure S2). Tryptophan is a pivotal precursor for indole alkaloids in relation with AS enzyme function and found to be reduced in our research. It was interesting that *AS* expression, however, was activated in this case. We interpret this result that the combined action of ET and MeJA simultaneously promotes the indole alkaloid and phenolic pathways, whilst tryptophan was rapidly consumed for indole alkaloid synthesis (Choi et al., 2004; Chung et al., 2007). Phenylpyruvic acid and *p*-hydroxyphenylpyruvic acid are precursors of L-phenylalanine and L-tyrosine, respectively, essential for C6C3 and C6C3C6 biosynthesis (Mustafa and Verpoorte, 2007). The *p*-hydroxyphenylpyruvic acid largely decreased in our research under the combined action of ET and MeJA, interpreting globally inhibited levels of C6C3C6 compounds. The gene expression level of *PAL* was both elevated obviously in this study, leading to the conversion of phenylalanine into cinnamic acid, which is a precursor for several phenolic compounds such as cinnamic acid, p-coumaric acid, caffeic acid, naringenin, and syringlc acid. The expression of C4H responsible for the hydroxylation at the C-4 position of cinnamic acid to form *p*-coumaric acid significantly increased, thereby contributing to the C6C3 compounds synthesis (Hotze et al., 1995).

## Conclusions

In conclusion, we report a comprehensive profiling analysis of phenolic compounds in response to the combinational action of ET and MeJA. Application of a widely non-targeted metabolomics method facilitated the identification of a total of 34 phenolic compounds and 7 intermediates for biosynthesis of phenolic compounds and indole terpenoid alkaloids (TIAs) using LC-MS/MS. Gene-to-metabolite networks were drawn up by searching for correlations between the expression profiles of 5 gene tags and the accumulation profiles of 41 metabolite peaks. The network revealed that the different branches of phenolic compounds biosynthesis and various other metabolic pathways are tightly regulated by ET-MeJA combination. Especially, the ET-MeJA cross-talk mainly promote C6C1- and C6C3-type, whilst inhibit C6C3C6-type phenolic compounds. Thus, this study provides an insight into the controlling mode of ET-MeJA combination on phenolic metabolism in *C. roseus* leaves. However, the underlying mechanisms in this induction process remain to be unraveled.

## Materials and methods

### Chemicals

All chemicals were of analytical reagent grade. Gradient grades of methanol, acetonitrile and acetic acid were purchased from Merck Company, German (http://www.merck-chemicals.com). Water was doubly deionized with a Milli-Q water purification system (Milford, MA, USA). Ethephon used to release ET and methyl jasmonate (MeJA) were both obtained from Sigma-Aldric (St. Louis, MO, USA).

### Plant material, growth conditions, and sample preparation

*C. roseus* seeds were planted in pots containing perlite and kept moistened until the seeds had germinated, and then irrigated with 1/2 strength Hoagland's solution (Pan et al., 2015). On the basis of process conditions screened, the concentrations of ethephon and MeJA used for treatment were 30 μM and 150 μM, respectively. Seedlings were used for treatments 30 d after cultivation with roots subjected to the 1/2 strength Hoagland's solution (control, T1), or plus 30 μM ethephon alone (T2) or 30 μM ethephon + 150 μM MeJA (T3). The total 30 individual plants were randomly selected and equally subjected into the conditions of T1, T2, and T3. The experiments were biologically repeated three times. The leaves of plants were harvested, 3 d after treatment for analysis of phenolic compounds and 4 h after treatment for analysis of gene expressions.

### LC-ESI-QTOF/MS analysis of phenolic compounds

For phenolic compound analysis, leaves were pulverized by grinding instrument (MM 400, Retsch, GmbH, Haan, Germany), and 50 mg tissue aliquots were extracted with 1.0 mL 70% aqueous methanol containing 0.1 mg/L lidocaine for water-soluble metabolites at 4°C overnight with three times vortexing. The extracts were clarified by centrifugation, combined, evaporated, and then filtered through 0.22 μm nylon membranes (SCAA 104; ANPEL http://www.anpel.com.cn/) before LC-MS analysis. Samples were analyzed using a liquid chromatography (LC) system coupled to a (QTOF) tandem mass spectrometer via electrospray ionization (ESI) interface (Agilent 6520) (Agilent Technologies, Santa Clara, CA, USA). Sample extracts were separated through a reversed phase on a Shim-pack LC column (VP-ODS C_18_ pore size 5.0 μm, 2^*^150 mm). The mobile phase consisted of solvent A and B. Solvent A contained 0.04% acetic acid in water, and solvent B 0.04% acetic acid in acetonitrile. The following gradient was used with a flow rate of 0.5 mL/min: 0–20 min, 5%B–95%B; 20–22.1 min: 95%B–5%B; 22.1–28 min: 5%B–5%B. Blank measurement with the initial solvent was carried out after each HPLC run. Injection volume and column temperature were set to 5 μL and 40°C, respectively. The optimized parameters for positive ion electrospray were as follows: capillary temperature of 350°C; curtain gas pressure of 40 psi; capillary voltage of 3500 V, fragmentation voltage of 135 V. The instrument was tuned prior to each batch run. A full-scan ranging between 50–1000 m/z was conducted with a scan time of 1 s and an interscan delay of 0.1 s in centered mode. The peak detection and matching were performed by Mass Hunter Qualitative (MHQ version B.03.01) and Mass Profiler Professional (MPP, version B.02.01) (Both from Agilent Technologies, Santa Clara, CA, USA). Metabolic features with mass, retention time, and abundance were obtained.

### RNA isolation and quantitative real time PCR analysis

For gene expression analysis, the total leaf RNA was extracted by TRIZOL reagent (Invitrogen). DNA contamination was removed using Dnase I following the instructions provided by the manufacturer (TaKaRa, Japan). DNA and RNA purity were observed using 1% agarose gel electrophoresis and RNA concentration was determined using a Nanodrap spectrophotometer (Thermo). cDNA was synthesized from total RNA (2 μg) using ReverTra Ace QPCR RT Kit (TOYOBO, Japan) according to the manufacturer's instructions, using oligo (dT) as the primer. qRT- PCR analysis using cDNA as template and gene-specific primers was performed using a SYBR Premix Ex Taq (TaKaRa, Japan) with an initial denaturation at 95°C for 30 s, followed by 35 cycles at 94°C for 30 s, 56°C for 30 s and 72°C for 30 s. Amplification, detection, and data analysis were carried out on a Rotor-Gene 6000 real-time rotary analyzer (Corbett Life Science, Sydney, Australia). The primers used were 5′-GCG AAC ATT TGC AGA TCC AT-3′ and 5′-GGC CGA TTT GTT ATT GTT CC-3′ for *AS*; 5′-GGC CAC CAA GAT GAT CGA-3′ and 5′-CAA TGG CCA ATC TTG CAT TG-3′ for *PAL*; 5′-GCC GAT TCT CTG TAT CAC TAT C -3′ and 5′-ATG ATT AAA ATG ATC TTG GCT TT-3′ for *C4H*; 5′-CGA TTT GTT GAA ATT GCA GAC G-3′ and 5′-ATT GCA GAC GAT CGT TTA ACT C-3′ for *CM*; 5′-ATT GCA GAC GAT CGT TTA ACT C-3′ and 5′-TTC CTC GGT CAA ACA TTT CG-3′ for *ICS*; (from ExPlant Technologies B.V.) (Table S1). These were repeated three times for each sample to ensure the reproducibility of results. Ribosomal protein subunit 9 (*Rsp9*) 5′-GAG GGC CAA AAC AAA CTT GA-3′ and 5′-CCC TTA TGT GCC TTT GCC TA-3′ was used as an internal control to evaluate all *C. roseus* plants.

### Statistical analysis

Metabolic pathways were performed in the Metaboanalyst web portal (http://www.metaboanalyst.ca) and MBRole (http://csbg.cnb.csic.es/mbrole). The pathways of metabolites were carried out on database sources including the KEGG (http://www.genome.jp/kegg/) to identify the top affected metabolic pathways and facilitate further metabolites interpretation. The metabolites and corresponding pathways were imported into Cytoscape software (v. 3.1.0) for visualization of the network models. The intersection of the networks was done using the advanced network merge function in Cytoscape. Pearson's correlation coefficients were calculated between metabolites and genes by SPSS 17.0. The Student's *t*-test and Tukey's test were used for mean value comparison. A total of 34 phenolic compounds were used for hierarchical clustering analysis by R (http://www.r-project.org/) to analyze phenolic profiles in response hormones.

## Author contributions

Conceived and designed the experiments: ZT; Performed the experiments: YL and YW; Analyzed the data: ZZ and JL; Wrote the paper: TE and JL.

### Conflict of interest statement

The authors declare that the research was conducted in the absence of any commercial or financial relationships that could be construed as a potential conflict of interest.

## Abbreviations

TIAs: terpenoid indole alkaloids
ABA: abscisic acid
ET: ethylene
MeJA: methyl jasmonate
PAL: phenylalanine ammonia lyase
CM: chorismate mutase
ICS: isochorismate synthase
AS: anthranilate synthase
C4H: cytochrome P450 hydroxylation
5.6-D-7,8,2,3,4-PF: 5,6′-dihydroxy-7, 8,2′,3′, 4′-Pentamethoxyflavone
Pro acid-*O*-hexoside: Protocatechuic acid-*O*-hexoside
Q-3-*O*-rhamnoside: Quercetin-3-*O*-rhamnoside
LC-MS/MS: liquid chromatography tandem mass spectrometry
ESI: electrospray ionization.
