# Supplementary figures and images for: The Combined Effects of Ethylene and MeJA on Metabolic Profiling of Phenolic Compounds in Catharanthus roseus Revealed by Metabolomics Analysis
Source: Front Physiol. 2016 Jun 7;7:217. doi: 10.3389/fphys.2016.00217 (PMC4895121; doi:10.3389/fphys.2016.00217)

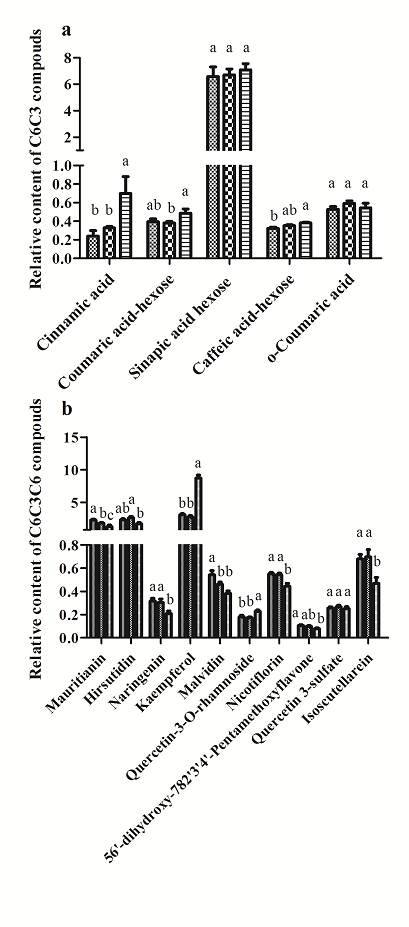

Supplement: Supplementary file 4 [file Image1.PNG]

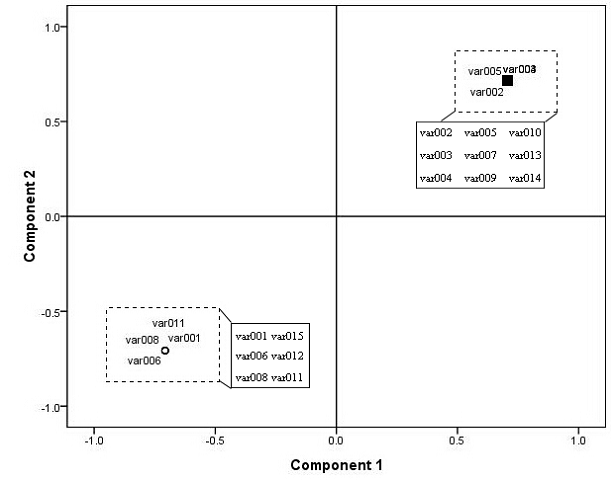

Supplement: Supplementary file 5 [file Image2.PNG]
